# The efficacy of an anatomy and ultrasonography workshop on improving residents’ confidence and knowledge in regional anesthesia

**DOI:** 10.1186/s12909-023-04653-y

**Published:** 2023-09-14

**Authors:** Derek J. Harmon, Christy K. Boscardin, Neal H. Cohen, Matthias R. Braehler

**Affiliations:** 1 Division of Anatomy, Department of Biomedical Education and Anatomy, The Ohio State University College of Medicine, 320 W 10th Ave. B104 Starling Loving Hall, Columbus, OH 43210 USA; 2 Department of Medicine and Anesthesia, University of California San Francisco, San Francisco, CA USA; 3 Department of Anesthesia and Perioperative Care and Medicine, University of California San Francisco, San Francisco, CA USA

**Keywords:** Medical education, Graduate medical education, Regional anesthesia, Ultrasound, Anatomy, Ultrasound guided regional anesthesia, Anesthesiology, Residency

## Abstract

**Background:**

Ultrasound Guided Regional Anesthesia (UGRA) has become the standard for regional anesthesia practice, but there is not a standardized educational approach for training residents. The objective of this study was to evaluate the efficacy of an UGRA workshop utilizing the theoretical framework of embodied cognition for anesthesiology residents.

**Methods:**

A workshop was developed consisting of didactics, scanning training on standardized patients (SPs) and anatomy reviews on prosected cadavers that focused on the most common UGRA procedures for the upper and lower extremity. At the beginning of the workshop and at the end of the workshop residents completed pre-test and pre-confidence surveys, as well as post-test and post-confidence surveys, respectively to assess the impact of the workshop.

**Results:**

39 residents (100% of the possible residents) participated in the workshop in 2019. Residents’ confidence in identifying relevant anatomy for the most common UGRA procedures significantly increased in 13 of the 14 measurements. Residents’ knowledge gain was also statistically significant from the pre-test to post-test (20.13 ± 3.61 and 26.13 ± 2.34; p < .0001). The residents found the course overall to be very useful (4.90 ± 0.38) and in particular the cadaveric component was highly rated (4.74 ± 0.55).

**Conclusions:**

In this study, we developed a workshop guided by the embodied cognition framework to aid in shortening the overall learning curve of UGRA for anesthesiology residents. Based on our results this workshop should be replicated by institutions that are hoping to decrease the learning curve associated with UGRA and increase residents’ confidence in identifying the relevant anatomy in UGRA nerve blocks.

**Supplementary Information:**

The online version contains supplementary material available at 10.1186/s12909-023-04653-y.

## Background

Ultrasound guided regional anesthesia (UGRA) has increased rapidly in popularity over the previous two and a half decades since a supraclavicular approach to the brachial plexus was first described [1–3]. UGRA has numerous benefits including reduced onset time of anesthesia, increased success rate, lower costs, reduced need for local anesthetics, and reduced risk of complications [3]. As a result of these benefits, UGRA has become the standard of care over alternative methods. Training in UGRA has therefore become an integral part of the educational competence for anesthesia residents. Competency in UGRA requires both cognitive and procedural skills [strengthened via thoughtful training experiences], including accurate interpretation of sonographic anatomy, basic principles of scanning, consistent needle imaging, appreciation of accurate local anesthetic spread, and reduced unintentional probe movement are critical in order to achieve competence in UGRA [4]. These competencies match well with the recommendations for the scope of practice and suggestions on developing a teaching curriculum for UGRA education published by the American Society of Regional Anesthesia (ASRA) [5]. Various studies have presented different approaches to UGRA workshops including low-fidelity simulators [6], computer-based simulators [7], and a needle visualization simulator [8], but there has been no standardized educational approach that attempts to directly target the anatomical knowledge gap.

In addition to the steep learning curve required to become proficient in ultrasonography, there is a robust field of literature that highlights the limited gross anatomy knowledge of residents entering their graduate medical education as a significant educational gap (GME) [9–11]. In a previous study [12], a group of residency directors, residents, and fourth year medical students were surveyed to measure their perceived anatomical competence (fourth year medical students and residents) or their perceived anatomical competence of their residents (residency program directors). All three groups from the study suggested potential improvements in anatomy education including: more cadaveric dissection in medical school and residency, more consistent teaching of anatomy for clinical practice, more workshops that review relevant anatomy, and better anatomical integration with the teaching of other subjects during medical school.

Development of a UGRA workshop requires a thoughtful design that can provide learners the ability to practice the three key integrated components (hand-eye coordination of the probe, anatomical image interpretation, and accurate needle placement). Embodied cognition provides a useful theoretical framework for understanding learning processes involving handheld devices that produce live imagery like ultrasonography and can serve as a guide for designing a workshop curriculum to teach UGRA to anesthesiology residents.

### Embodied cognition

Embodied cognition is a theoretical approach that describes the relationship between our body, mind, and our environment in cognitive processes [13–17]. One early investigation of embodied cognition [13] led to the framework that was called the “perceptual symbol” system. This framework argues that symbols are created by all the incoming sensory information from an individual’s given experience, which are then encoded by the brain and stored within our sensory centers. The key principles of embodied cognition that came out of the perceptual symbol study [13] quickly expanded into educational research and curricular development.

Clinical medicine often requires procedures involving physical manipulation of a patient (e.g., physical exam) and/or a device (e.g., ultrasonography) along with immediate interpretation of the findings of these evaluations. This intimate connection of the body, mind, and environment illustrates why embodied cognition is a worthwhile framework to use when developing a clinical training workshop on UGRA. The clinician must be able to coordinate the probe with their hand in the correct plane of the targeted tissue, the anatomy presented on screen must be interpreted quickly and accurately, and the interventional device (e.g., block needle), must be introduced at the correct angle and depth. Developing a workshop through the lens of an embodied cognition framework could provide deliberate practice for the learners that may better prepare them for their UGRA clinical practice.

In this study, we developed a workshop guided by the embodied cognition framework to increase the knowledge and confidence of anesthesiology residents when conducting UGRA procedures. The workshop provided learners with the hands-on opportunity of scanning live models with real time feedback from anesthesiology clinical faculty along with a thorough neuroanatomy review of the limbs on fully prosected cadaveric donors led by anatomists. By providing the hands-on ultrasound experience along with the anatomical tissue visualization in-situ, we aimed to strengthen the motor-action neural circuit involved in embodied cognition in our trainees. The workshop was specifically designed to help connect the three-dimensional structure to the two-dimensional image as well as solidify hand-eye coordination through practice and feedback. In this study, we describe the development, implementation, and preliminary evaluation of this novel UGRA workshop for residents.

## Methods

As a required part of resident education, a regional anesthesia workshop was organized and conducted twice in May of 2019. The workshop was offered to all PGY-3 and PGY-4 residents in anesthesiology, and all residents were randomly assigned to a workshop date in May. The PGY-3 residents were taking this workshop prior to the start of their required regional anesthesia rotation as a part of their residency training. The PGY-4 residents were taking this workshop for the second time as they had all participated in the same workshop the previous year prior to the start of their regional anesthesia rotation. The workshop was offered over two days in in order to maintain a low resident-to-faculty ratio. The PGY-4 residents were invited to participate in the workshop for a second year due to high demand from this cohort to repeat the workshop prior to completing their residency.

The workshop began with residents being assigned a random number to track their assessments as well as to assign them to laboratory groups at the beginning of the workshop, followed by the residents taking the pre-test (knowledge-based test) and the pre-survey (confidence survey). Confidence (self-efficacy) was chosen as a measurement for this study, as it has previously shown to directly correlate with performance [18–21]. Residents then received three didactic lessons, which focused on upper extremity neuromusculoskeletal gross anatomy (delivered by an anatomist), basics of ultrasonography, and UGRA for the upper extremity (both ultrasound-related lectures were delivered by an anesthesiologist/UGRA expert). Following the didactics, the residents were divided into two groups, the “anatomy group” and the “scanning group”. For the “anatomy group” two cadaveric donors were dissected previously by an anatomist specifically for this workshop in order to provide the most direct visualization of the relevant neuromusculoskeletal anatomy for the upper and lower extremity. The dissections focused not only on the nerves that would be blocked in each of the UGRA procedures but provided the necessary context of the adjacent structures at risk of puncture or of being blocked in addition to the target structures. These prosections provided a thorough overview of both the relevant anatomy, as well as an opportunity to address the potential risks for each block. The anatomy group received a neuromusculoskeletal anatomy review of the upper extremity focused on the relevant UGRA blocks. The scanning group was divided evenly and paired with an anesthesiology faculty (3 residents to 1 faculty) and a standardized patients (SPs) for the scanning sessions. The anesthesiology faculty followed a standardized approach throughout the workshop by first demonstrating to the students how to conduct an optimal scan for a particular UGRA block (e.g., interscalene block) and then asking all of the residents to practice scanning that block until they were comfortable. Faculty were monitoring and providing formative feedback to the residents about their motor skills related to using the probe as well as the quality of their ultrasound imaging for each scan. The interscalene, supraclavicular, infraclavicular, axillary, forearm, and wrist UGRA upper extremity blocks were covered in both groups. After 45 min, the groups switched, and the sessions were repeated. Following the upper limb sessions, the residents came back together for another didactic lesson focused on lower extremity neuromusculoskeletal gross anatomy (delivered by an anatomist), and UGRA for the lower extremity (delivered by an anesthesiologist/UGRA expert). Then the residents divided into anatomy and scanning groups to cover the lower extremity material. The anatomy group received a neuromusculoskeletal anatomy review of the lower extremity focused on the relevant UGRA blocks. The fascia iliaca, femoral, adductor canal, saphenous, sciatic, popliteal, and ankle UGRA lower extremity blocks that were covered in both groups. After 45 min, the groups switched, and the sessions were repeated. After the final lab sessions, the residents completed the post-test, the post-survey, and course evaluation. The pre-test and post-test consisted of thirty multiple choice knowledge-based questions, developed by a team of medical educators, regional anesthesiologists, and anatomists (Appendix A). Content validity was established in the knowledge-based tests by the two authors, who are experts in the field of anatomy and ultrasonography, respectively. This led to the determination that assessing residents’ confidence in identifying (and thus being able to manipulate a probe to locate) the relevant neuroanatomy for each block provided an appropriate measurement that corresponded with the objectives of the workshop.

We evaluated this workshop by measuring the learners’ anatomical and ultrasonography knowledge along with their confidence at identifying the relevant neuroanatomy in the most common upper and lower extremity UGRA blocks. These two assessments were chosen as the integration of hand-eye coordination involved in ultrasonography and accurate anatomical image interpretation are critical requirements for UGRA. In addition, confidence (self-efficacy) was chosen as the measure for the survey as it has been shown previously to correlate with performance [18–21].

The pre-confidence and post-confidence survey consisted of fourteen Likert-type questions on a 1–5 scale (1 = not confident to 5 = very confident) and focused on the residents’ confidence in identifying the relevant neuroanatomical tissues associated with UGRA of the upper and lower extremities through ultrasound imaging (Appendix B). As tests of differences on fourteen a priori hypotheses were conducted, a Bonferroni adjusted alpha levels of 0.0036 per test (0.05/14) was used for statistical analysis. In the post-survey residents were asked to evaluate the educational value of the workshop (Likert-scale 1–5; 1 = not valuable at all, 5 = very valuable) and to provide any comments.

Paired sample t-tests were used to compare the pre-test and post-test scores and independent samples t-tests were used to compare the PGY3 and PGY4 residents’ pre-test and post-test scores. Wilcoxon signed-ranks test was used to test the mean ranks difference between the pre-survey and the post-survey confidence ratings. Knowledge tests and confidence surveys were both stratified by PGY and combined as a single group for analysis. Internal consistency was assessed with Cronbach alpha according to the criteria published previously [22]. The University of California San Francisco Institutional Review Board approved this study (IRB# 19-28188).

## Results

The entire population of PGY-3 and PGY-4 residents at UCSF (n = 39, 100%) participated in the workshop. Twenty-five (64%) residents participated in the first workshop (fourteen (56%) PGY-3 and eleven (44%) PGY-4 residents). Fourteen residents (36%) participated in the second workshop (eight (57%) PGY-3 and six (43%) PGY-4 residents). All participants completed the assessments and surveys. Before the workshop residents had the highest level of confidence in identifying the femoral nerve in a femoral nerve block (3.63 ± 1.04) and the tibial/common peroneal nerves in the popliteal nerve block (3.53 ± 0.99), while the tibial nerve in an ankle block (1.71 ± 0.89) and the cords of the brachial plexus in an infraclavicular block (2.42 ± 0.99) had the lowest confidence rating by the residents. At the end of the workshop, the confidence in identifying the femoral nerve in a femoral nerve block (4.13 ± 0.86) and the tibial/common peroneal nerves in a popliteal nerve block (4.08 ± 0.74) had the highest ratings, while the tibial nerve in an ankle block (3.23 ± 1.04) and the cords of the brachial plexus in an infraclavicular block (3.38 ± 0.88) had the lowest confidence rating by the residents. Cronbach alpha coefficient was 0.93 for the survey and 0.75 for the test, suggesting that both instruments had acceptable internal validity [22]. Evidence for construct validity was obtained through the results of the internal consistency (reliability) assessment of the knowledge and confidence tools.

**Fig. 1:**
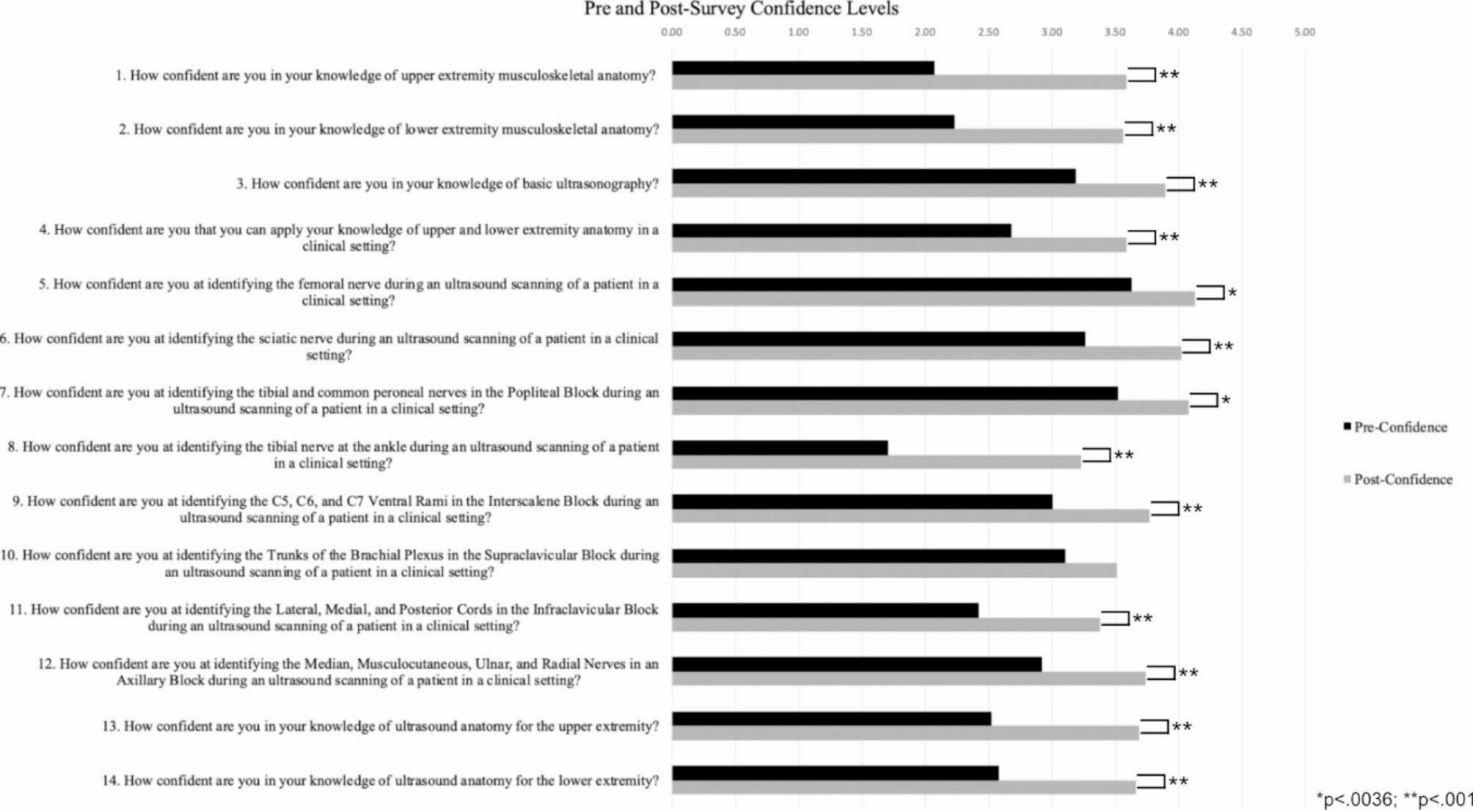
Comparison of Pre- and Post-Confidence Surveys for all 39 PGY-3 and PGY-4 Anesthesia Residents. The alpha level was adjusted through the Bonferroni correction, which adjusted the significance level to 0.0036. Thirteen of the fourteen confidence statements had statistically significant increases following the workshop. *p < .0036; ** p < .001

**Fig. 2:**
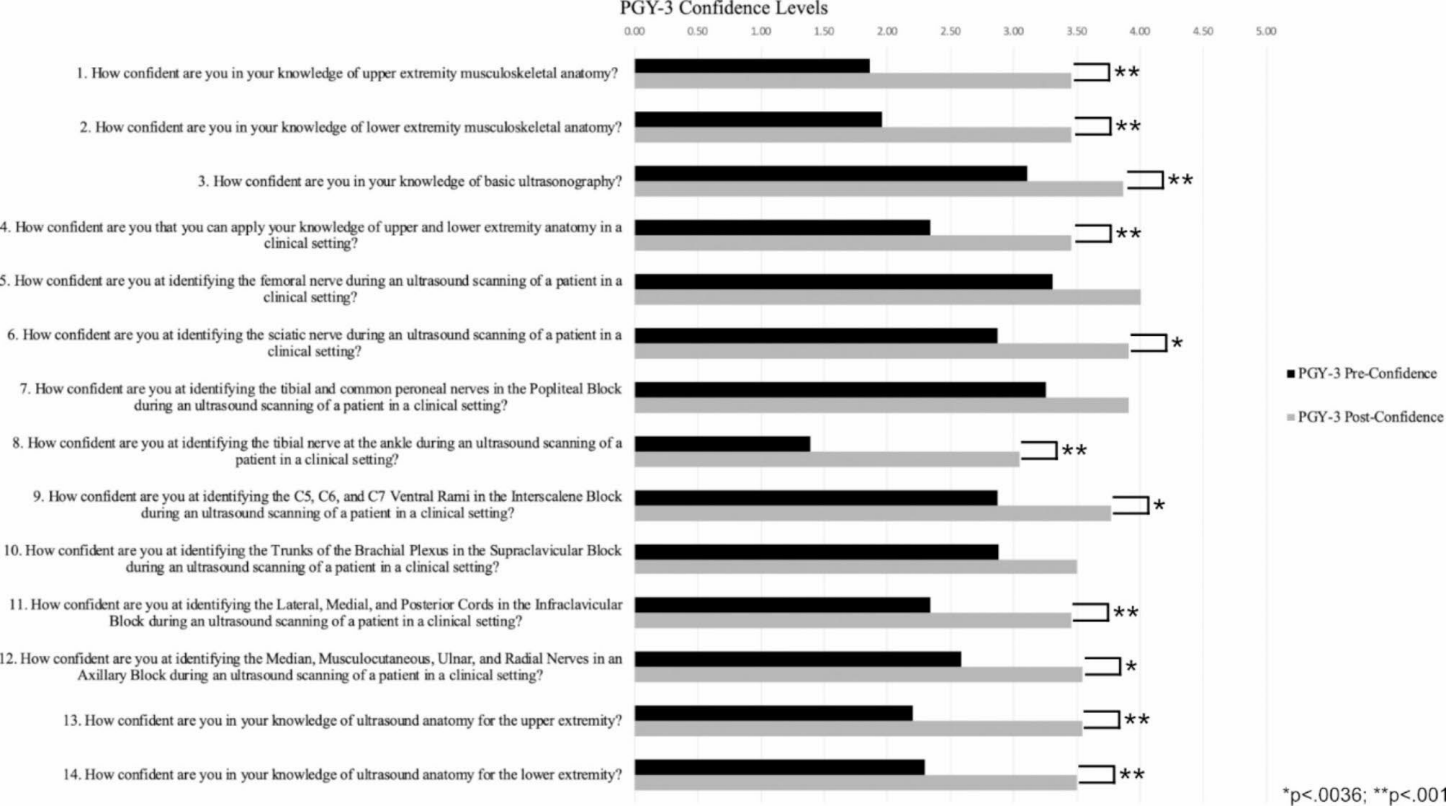
Comparison of Pre- and Post-Confidence Surveys for all 22 PGY-3 Anesthesia Residents. The alpha level was adjusted through the Bonferroni correction, which adjusted the significance level to 0.0036. Eleven of the fourteen confidence statements had statistically significant increases following the workshop. *p < .0036; ** p < .001

**Fig. 3:**
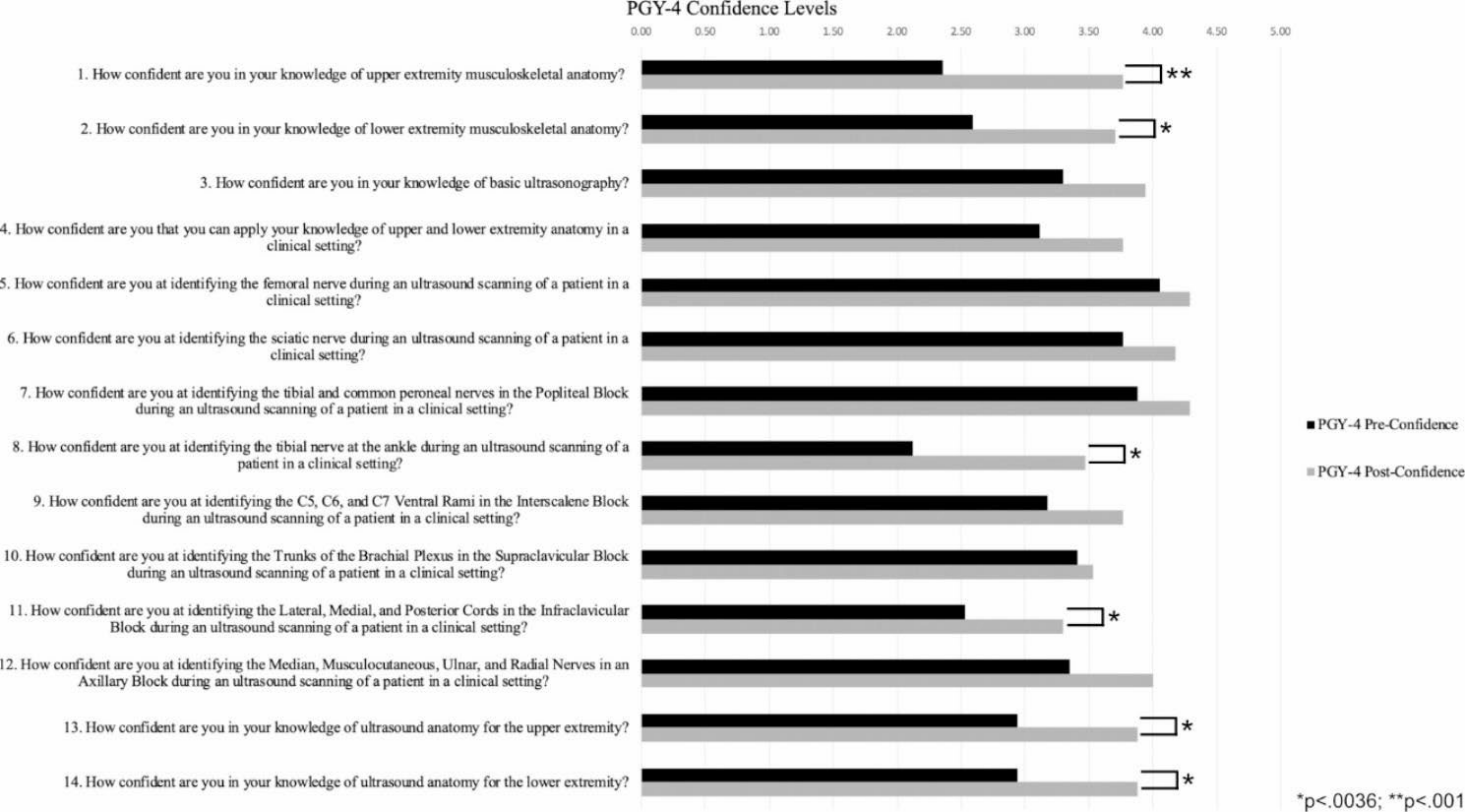
Comparison of Pre- and Post-Confidence Surveys for all 17 PGY-4 Anesthesia Residents. The alpha level was adjusted through the Bonferroni correction, which adjusted the significance level to 0.0036. Six of the fourteen confidence statements had statistically significant increases following the workshop. *p < .0036; ** p < .001

There was a statistically significant (p < .0036) increase in residents’ confidence level for thirteen of the fourteen confidence statements that were evaluated (Fig. 1). Stratifying the data based on PGY, eleven of the fourteen confidence statements had statistically significant increases for PGY-3 residents (Fig. 2) and statistically significant increases for PGY-4 residents were found in six of the fourteen statements (Fig. 3).

There was a statistically significant increase in residents’ knowledge from the pre-test to post-test (20.13 ± 3.61 and 26.13 ± 2.34; p < .0001). Stratifying the test data based on PGY, there was a statistically significant difference in scores for PGY-4 residents compared to PGY-3 residents for both the pre-test (22.47 ± 2.94 and 18.32 ± 3.03; p < .001) and post-test (27.35 ± 2.23 and 25.18 ± 1.99; p = .003) respectively. There was a statistically significant increase from the pre-test to post-test scores for PGY-3 residents (p < .0001) and for PGY-4 residents (p < .0001) at the end of the workshop.

The post-survey also included questions evaluating the workshop. The residents found the course overall to be very useful (4.90 ± 0.38), the cadaveric component to be very useful (4.74 ± 0.55), and the workshop overall to be very useful in comparison to their prior educational experiences (4.87 ± 0.41). All results from the workshop evaluation are in Table 1.

**Table 1:** Resident Evaluation of Workshop

Evaluation Question	Mean	SD
How clear were the course objectives?	4.77	0.485
How appropriate was the content to your level of learning?	4.87	0.409
How effective was this course in enhancing your knowledge of upper extremity anatomy?	4.79	0.522
How effective was this course in enhancing your knowledge of lower extremity anatomy?	4.87	0.409
How useful did you find the course overall?	4.90	0.384
How useful was the lecture component of the course?	4.72	0.510
How useful was the standardized patient component of the course?	4.67	0.701
How useful was the cadaveric lab component of the course?	4.74	0.549
How useful was this course in comparison to your prior educational experiences?	4.87	0.409

## Discussion

In this study, we assessed the implementation of an anatomy and ultrasound workshop that targeted skills required to become proficient in ultrasonography as described by ASRA [5] including: (1) understanding device operation, (2) image optimization, (3) image interpretation, and (4) visualization of the needle insertion. In order to target these skills, the workshop utilized embodied cognition as the theoretical framework due to the coordination required with the plane of the ultrasound probe and the accurate anatomical image interpretation. Based on previous authors’ [23] perspective on embodied cognition taxonomy, we aimed to develop a curriculum that involved a high-level of body engagement focused on a particular task. We believed that this specific embodied cognition perspective would create a strong motor-action neural circuit in the anesthesiologists when conducting UGRA. By developing the workshop around this framework, we believe this was a major factor that explains the significant increases in resident confidence of identifying relevant neuroanatomical tissues associated with UGRA of the upper and lower extremities.

The effectiveness of this workshop was assessed through both the knowledge-based and the confidence level assessments of residents. By the end of the workshop, residents rated their confidence in identifying relevant neuroanatomy structures for UGRA upper and lower extremity scans to be significantly higher than at the beginning of the workshop. The statistically significant increase of scores on the knowledge-based assessments indicates an increase in resident knowledge of both ultrasonography and neuroanatomy for the upper and lower extremities. These measurements could provide direct evidence to the efficacy of this workshop at increasing proficiency of UGRA based on the recommendations of the ASRA [5].

Based on the findings from this approach to train anesthesia residents in UGRA, future iterations of this workshop will include an objective structured clinical examination (OSCE)-type assessment as a part of the scanning group sessions. The next cohort of anesthesiology residents that participate in the workshop will be recruited to complete the same knowledge-based and confidence assessments six months following the workshop to measure retention. Similar workshops focused on trunk blocks and transesophageal echocardiography (TEE), where residents will be able to practice scans and visualize the relevant anatomy on cadaveric specimens are in development.

The development of this workshop required collaboration between anesthesiologists and anatomists in order to develop a curriculum that logically combined their respective disciplines. Replication of this workshop is ideally suited for institutions with access to a cadaveric laboratory, anatomy faculty, and clinical anesthesiology faculty due to the required cadaveric dissections to emphasize the relevant neuroanatomy of the upper and lower extremities as well as development of didactic lessons to focus on the most relevant clinical topics. Although this workshop was tailored to anesthesiologists, integrating cadaveric based gross anatomy education into residencies has shown to be successful in numerous other fields [24–32]. Residencies that rely heavily on robust gross anatomy knowledge may benefit from similar workshops.

This study had several limitations. It was conducted at a single institution (UCSF) and focused on a single medical discipline that utilizes ultrasonography (anesthesiology). Another limitation is that while we included all PGY-3 and PGY-4 anesthesiology residents, the overall number of participants (n = 39) was relatively low. The authors also acknowledge that in order to understand fully the findings from this study and to draw broader conclusions there is a need to validate the survey instrument and to assess the reliability at different institutions, particularly at other anesthesiology residency programs.

## Conclusion

In this study, we developed a workshop guided by the embodied cognition framework to increase the knowledge and confidence of anesthesiology residents when conducting UGRA procedures. Our results showed a significant increase in confidence for both PGY-3 and PGY-4 residents in identifying the relevant anatomy in UGRA blocks as well as in their ultrasound and anatomy knowledge. The findings from this study support the recommendation that other training programs consider implementation of similar approaches to teaching UGRA with the goal of shortening the learning time and optimizing education.

### Electronic supplementary material

Below is the link to the electronic supplementary material.

Supplementary Material 1

Supplementary Material 2

## Data Availability

The datasets used and/or analyzed during the current study available from the corresponding author on reasonable request.

## Abbreviations

ASRA: American Society of Regional Anesthesia
ESRA: European Society of Regional Anesthesia and Pain Therapy
GME: Graduate Medical Education
OSCE: Objective Structured Clinical Examination
PGY: Post Graduate Year
SP: Standardized Patient
TEE: Transesophageal Echocardiography
UGRA: Ultrasound Guided Regional Anesthesia

## References

[1] Kapral S, Krafft P, Eibenberger K, Fitzgerald R, Gosch M, Weinstabl C (1994). Ultrasound-guided Supraclavicular Approach for Regional Anesthesia of the Brachial Plexus. Anesth Analgesia.

[2] Martinelli SM, Monroe H, Coombs R, Miller N, Borstov A, Salo-Coombs V. Assessing the impact of a Regional Anesthesia Workshop on Anesthesiology residents’ perceived comfort in performing peripheral nerve blocks. J Educ Perioper Med. 2012;14. PMC4719547 27175392

[3] Chen XX, Trivedi V, AlSaflan AA, Todd SC, Tricco AC, McCartney CL, Boet S (2017). Ultrasound-guided Regional Anesthesia Simulation Training: a systematic review. Reg Anesth Pain Med.

[4] Filho GRDO, Helayel PE, Conceicao DB, Garzel IS, Pavei P, Ceccon MS. (2008). Learning Curves and Mathematical Models for Interventional Ultrasound Basic Skills. Anesthesia & Analgesia. 2008;106:568–573. 10.1213/ane.0b013e3181605412 18227318

[5] Sites BD, Chan VW, Neal JM, Weller R, Grau T, Koscielniak-Nielsen ZJ, Ivani G (2010). The American Society of Regional Anesthesia and Pain Medicine and the European Society of Regional Anesthesia and Pain Therapy Joint Committee Recommendations for Education and Training in Ultrasound-Guided Regional Anesthesia. Reg Anesth Pain Med.

[6] Niazi AU, Haldipur N, Prasad AG, Chan VW (2012). Ultrasound-guided regional anesthesia performance in the early learning period: effect of simulation training. Reg Anesth Pain Med.

[7] Woodworth GE, Chen EM, Horn JL, Aziz MF (2014). Efficacy of computer-based video and simulation in ultrasound-guided regional anesthesia training. J Clin Anesth.

[8] Lam NC, Fishburn SJ, Hammer AR, Petersen TR, Gerstein NS, Mariano ER (2015). A Randomized Controlled Trial evaluating the See, Tilt, Align, and rotate (STAR) Maneuver on Skill Acquisition for simulated ultrasound-guided interventional procedures. J Ultrasound Med.

[9] Fillmore EP, Brokaw J, Kochhar K, Nalin PM (2016). Understanding the current anatomical competence landscape: comparing perceptions of program directors, residents, and fourth-year medical students. Anat Sci Educ.

[10] Cottam WW (1999). Adequacy of medical school gross anatomy education as perceived by certain postgraduate residency programs and anatomy course directors. Clin Anat.

[11] Turney B (2007). Anatomy in a modern medical curriculum. Ann R Coll Surg Engl.

[12] Ahmed K, Rowland S, Patel V, Khan RS, Ashrafian H, Davies DC, Darzi A, Athanasiou T, Paraskeva PA (2010). Is the structure of anatomy curriculum adequate for safe medical practice?. Surgeon.

[13] Barsalou LW. (1999). Perceptions of Perceptual Symbols. Behav Brain Sci. 1999;22:637–660. 10.1017/s0140525x99002149 11301525

[14] Shapiro L (2010). Embodied cognition.

[15] Glenberg AM (2010). Embodiment as a unifying perspective for psychology. Wiley Interdisciplinary Reviews: Cognitive Science.

[16] Beilock S (2015). How the body knows its mind: the surprising power of the physical environment to influence how you think and feel.

[17] van der Schaaf M, Bakker A, Ten Cate O. When I say … embodied cognition. Med Educ. 2019 Mar;53(3):219–220. 10.1111/medu.13678 10.1111/medu.13678 30259543

[18] Mavis B (2001). Self-efficacy and OSCE performance among second year medical students. Adv Health Sci Educ.

[19] Opacic DA (2003). The relationship between self-efficacy and student physician assistant clinical performance. J Allied Health.

[20] Lane J, Lane A, Kyprianou A (2004). Self-efficacy, self-esteem and their impact on academic performance. Social Behav Personality: Int J.

[21] Brady-Amoon, Peggy, Jairo N, Fuertes (2011). Self‐Efficacy, Self‐Rated abilities, adjustment, and academic performance. J Couns Dev.

[22] Taber KS (2018). The use of Cronbach’s alpha when developing and reporting research instruments in science education. Res Sci Educ.

[23] Skulmowski A, Rey GD (2018). Embodied learning: introducing a taxonomy based on bodily engagement and task integration. Cogn Res Princ Implic.

[24] Jansen S, Cowie M, Linehan J, Hamdorf JM (2014). Fresh frozen cadaver workshops for advanced vascular surgical training. ANZ J Surg.

[25] Sharma G, Aycart MA, Najjar PA, van Houten T, Smink DS, Askari R, Gates JD (2016). A cadaveric procedural anatomy course enhances operative competence. J Surg Res.

[26] Kim SC, Fisher JG, Delman KA, Hinman JM, Srinivasan JK (2016). Cadaver-based Simulation increases Resident confidence, initial exposure to fundamental techniques, and May augment operative autonomy. J Surg Educ.

[27] Juo YY, Hanna C, Chi Q, Chang G, Peacock WJ, Tillou A, Lewis CE (2018). Mixed-method evaluation of a cadaver dissection course for general surgery Interns: an innovative Approach for filling the gap between gross anatomy and the operating room. J Surg Educ.

[28] Slieker JC, Theeuwes HP, van Rooijen GL, Lange JF, Kleinrensink GJ (2012). Training in laparoscopic colorectal surgery: a new educational model using specially embalmed human anatomical specimen. Surg Endosc.

[29] Jaswal J, D’Souza L, Johnson M, Tay K, Fung K, Nichols A, Landis M, Leung E, Kassam Z, Willmore K, D’Souza D, Sexton T, Palma DA (2015). Evaluating the impact of a canadian national anatomy and radiology contouring boot camp for radiation oncology residents. Int J Radiat Oncol Biol Phys.

[30] Darras KE, Spouge R, de Bruin A, Hu J, Guest W, Mar C, Hatala R, Hague C, Forster BB, Chang SD (2018). Development and evaluation of a competency-based anatomy rotation for diagnostic radiology residents during internship year: a canadian experience. Can Assoc Radiol J.

[31] Demars N, Compère V, Duparc F, Fourdrinier V, Dureuil B (2010). Contribution of the anatomy laboratory to the practical training of residents in regional anesthesia. Surg Radiol Anat.

[32] DeFriez CB, Morton DA, Horwitz DS, Eckel CM, Foreman KB, Albertine KH (2011). Orthopedic resident anatomy review course: a collaboration between anatomists and orthopedic surgeons. Anat Sci Educ.

