# Supplementary material for: The efficacy of an anatomy and ultrasonography workshop on improving residents’ confidence and knowledge in regional anesthesia
Source: BMC Med Educ. 2023 Sep 14;23:665. doi: 10.1186/s12909-023-04653-y (PMC10500924; doi:10.1186/s12909-023-04653-y)
Supplement: Supplementary file 1 — Supplementary Material 1 [file 12909_2023_4653_MOESM1_ESM.docx]

Appendix A – Knowledge Pre and Post-Test Assessment

Name:__________________________________________________________________ PGY:______________

Please circle the correct answer choice.

Question 1: Nerves blocked with a fascia iliaca block include the

1. Sciatic nerve
2. Femoral nerve
3. Lateral Femoral Cutaneous Nerve
4. A and B
5. A, B, C
6. B and C
7. All of the above

Question 2: In the diagram below, regional anesthesia of the area depicted by #1 can be accomplished with a block of the:

1. Radial nerve
2. Musculocutaneous nerve
3. Ulnar nerve
4. Median nerve
5. Coracobrachialis nerve


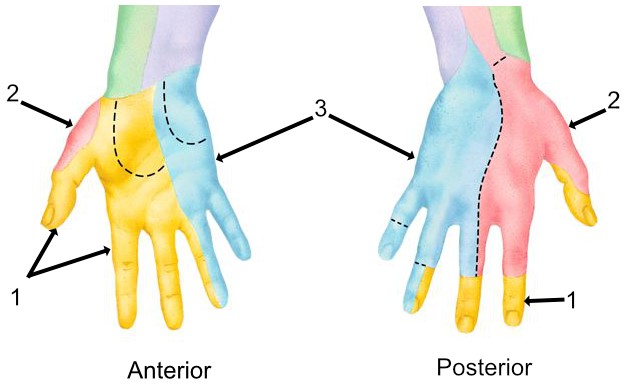


Question 3: Which structure accompanies the ulnar nerve in the distal forearm?

1. Radial artery
2. Ulnar artery
3. Medial branch of the median nerve
4. Adductor pollicis muscle
5. Palmaris longus tendon

Question 4: Which movements belong to the ive basic ultrasound probe maneuvers?

1. Gliding
2. Tilting
3. Skating
4. Probing
5. All of the above

Question 5: Branches of the femoral nerve anesthetized during an ankle block include:

1. Super icial peroneal nerve
2. Deep peroneal nerve
3. Tibial nerve
4. Saphenous nerve
5. Sural nerve

Question 6: Which are the branches coming off the posterior cord of the brachial plexus?

1. Axillary and long thoracic nerves
2. Median and musculocutaneous nerves
3. Ulnar and medial antebrachial nerves
4. Axillary and radial nerves
5. Median and radial nerves

Question 7: Which muscle usually needs to be penetrated in order to perform an adductor canal block?

1. Adductor longus muscle
2. Adductor brevis muscle
3. Adductor magnus muscle
4. Sartorius muscle
5. Vastus medialis muscle

Question 8: What does the term “Anisotropy” refer to?

1. Change of visibility of a nerve depending on the patient’s Body Mass Index
2. Change of visibility of a nerve depending on the tilt of the ultrasound probe
3. Change of visibility of a nerve depending on the depth of the target
4. Difference appearance of muscles depending on the number of muscle ibers
5. Worsening of the visibility of nerves with the use of certain anesthesia gases

Question 9: What is the most commonly used approach for ultrasound guided peripheral nerve blocks?

1. “No plane, no gain”
2. “Long axis, out of plane”
3. “Long axis, in plane”
4. “Short axis, out of plane”
5. “Short axis, in plane”

Question 10: Using the igure below, a block of the sural nerve would produce anesthesia of (select the appropriate letter of the igure):


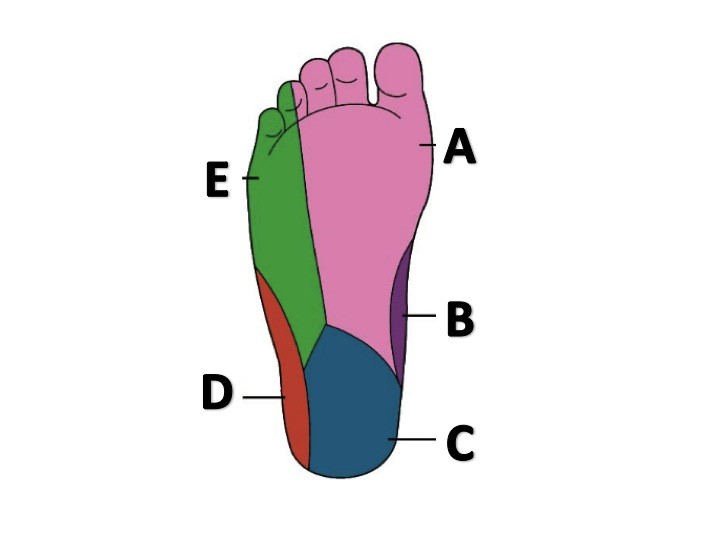


Question 11: Between which muscles does the brachial plexus travel in the interscalene approach?

1. Anterior scalene and sternocleidomastoid muscles
2. Anterior and middle scalene muscles
3. Middle and posterior scalene muscles
4. Anterior and posterior scalene muscles
5. Pectoralis major and minor muscles

Question 12: In the parasagittal view of the fascia iliaca block, the arrows here indicate muscle bellies of the:


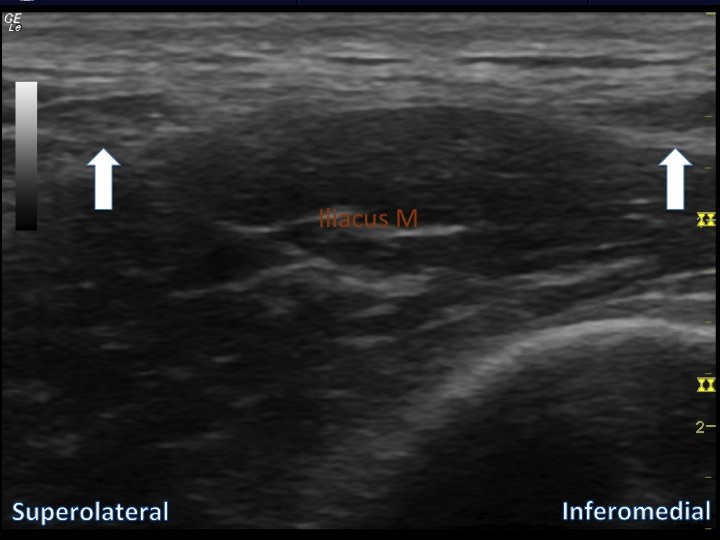


1. Transverse abdominis and internal oblique
2. Internal oblique and rectus femoris
3. Internal oblique and sartorius
4. Sartorius and pectineus
5. Sartorius and adductor brevis

Question 13: The white arrow here in the supraclavicular view indicates:


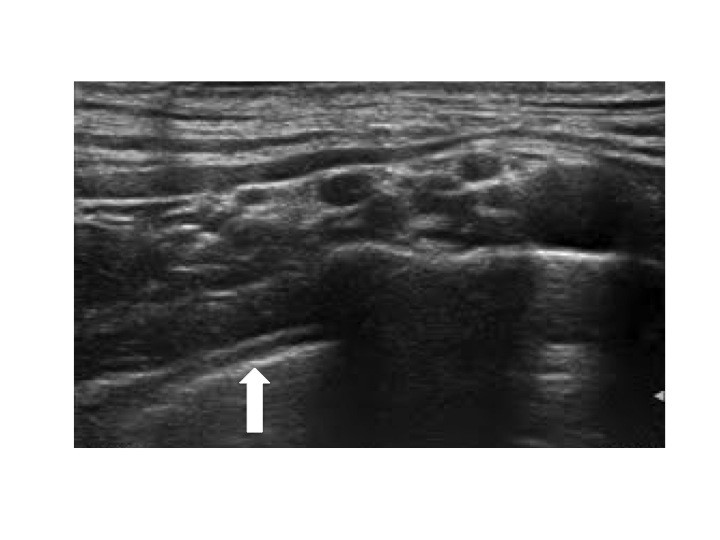


1. Coracoid process
2. First rib
3. Clavicle
4. Pleura
5. Conjoint tendon

Question 14: The phrenic nerve arises from the

1. Nucleus ambiguous
2. Sympathetic side chain
3. C1‐C2 nerve roots
4. C3‐C5 nerve roots
5. C6‐T1 nerve roots

Question 15: Between which muscles is the musculocutaneous nerve usually sandwiched in the axillary brachial plexus block?

1. Biceps and triceps muscles
2. Coracobrachialis and triceps muscles
3. Coracobrachialis and biceps muscle
4. Biceps and deltoid muscle
5. Latissimus dorsi and triceps muscle

Question 16: What does the hyperechoic structure behind the femoral artery represent?


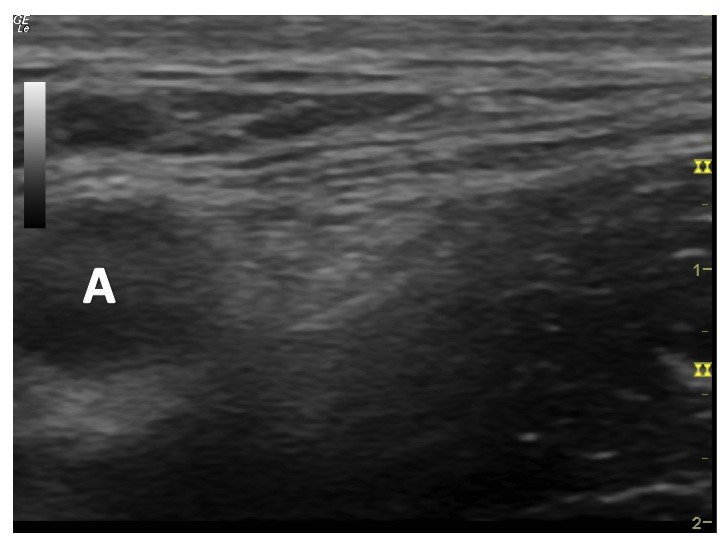


1. Deep femoral nerve branch
2. Saphenous nerve
3. Posterior branch of the obturator nerve
4. Lateral femoral cutaneous nerve
5. Acoustic enhancement artifact

Question 17: For a super icial cervical plexus block, the needle enters

1. Slightly below the mastoid process
2. Along the upper border of the clavicle
3. At the posterior border of the sternocleidomastoid muscle
4. Medial from the coracoid process
5. Below the cricoid cartilage

Question 18: Which muscles does the needle pass for an infraclavicular nerve block?

1. Deltoid and pectoralis major
2. Sternocleidomastoid and anterior scalene
3. Latissimus dorsi and serratus anterior
4. Teres major and teres minor
5. Pectoralis major and minor

Question 19: Which structure passes anterior to the anterior scalene muscle?

1. Subclavian vein
2. Subclavian artery
3. Roots of the brachial plexus
4. Sympathetic trunk
5. Stellate ganglion

Question 20: The recurrent laryngeal nerve:

1. lies anterior to the anterior scalene muscle
2. lies in the interscalene triangle
3. ascends posterior to the thyroid gland
4. emerges from behind the sternocleidomastoid muscle
5. is a branch of the cervical plexus of nerves

Question 21: Potential complications from the interscalene nerve block include:

1. Mydriasis
2. Miosis
3. Hoarseness
4. Inability to close their eyelid
5. A and C
6. B and C
7. C and D
8. All of the above

Question 22: The cords of the brachial plexus (medial, lateral, posterior) are named for their relationship to the:

1. Subclavian artery
2. Subclavian vein
3. Axillary artery
4. Axillary vein
5. Clavicle

Question 23: The skin on the lateral part of the forearm is innervated by the:

1. Musculocutaneous nerve
2. Median nerve
3. Ulnar nerve
4. Axillary nerve
5. Radial nerve

Question 24: The lumbar plexus is made up of nerves from:

1. L1 – L4
2. L2 – L4
3. L4 – S3
4. L1 – S4
5. S2 – S4

Question 25: The femoral nerve innervates the:

1. Hamstring muscles
2. Adductor muscles of the thigh
3. Quadriceps muscles
4. Medial malleolus
5. A and B
6. A, B, C only
7. C and D
8. All of the above

Question 26: Into which two branches does the sciatic nerve split?

1. Deep and super icial peroneal nerves
2. Saphenous and sural nerves
3. Tibial and common peroneal nerves
4. Femoral and obturator nerves
5. Posterior and anterior tibial nerves

Question 27: What is an appropriate motor response to nerve stimulation for a lumbar plexus block?

1. Isolated adductor twitch
2. Isolated hamstring twitch
3. Isolated quadriceps twitch
4. Dorso lection of the foot
5. Plantar lexion of the foot

Question 28: Which nerve is not a sensory nerve?

1. Deep radial nerve
2. Super icial radial nerve
3. Saphenous nerve
4. Lateral femoral cutaneous nerve
5. Supraclavicular nerve

Question 29: Which of the following is **not**  a contraindication for an interscalene brachial plexus block?

1. Patient’s age > 85 years
2. Infection at the puncture site
3. Contralateral phrenic nerve palsy
4. Severe COPD
5. Patient refusal to have a block

Question 30: Which nerve will most frequently be missed by any brachial plexus block?

1. Ulnar nerve
2. Musculocutaneous nerve
3. Axillary nerve
4. Lateral antebrachial cutaneous nerve
5. Intercostobrachial nerve
