# Supplementary material for: The efficacy of an anatomy and ultrasonography workshop on improving residents’ confidence and knowledge in regional anesthesia
Source: BMC Med Educ. 2023 Sep 14;23:665. doi: 10.1186/s12909-023-04653-y (PMC10500924; doi:10.1186/s12909-023-04653-y)
Supplement: Supplementary file 2 — Supplementary Material 2 [file 12909_2023_4653_MOESM2_ESM.docx]

**PRE SURVEY**

**PGY Year _______ Unique ID _____________________**

**(General attitudes toward anatomy education)**

**How important is it to you to learn anatomy outside of the operating room or clinic?**

1- Not important 2- Slightly important 3- Fairly important 4- Important 5- Very important

**What is the primary format in which you study anatomy currently (textbook, mobile app, videos, etc.)?**

**How satisfied are you with this method?**

1- Very dissatisfied 2- Dissatisfied 3- Neither 4- Satisfied 5- Very satisfied

**(Confidence in musculoskeletal regional anatomy and ultrasound knowledge)**

**How confident are you in your knowledge of upper extremity musculoskeletal anatomy?**

1- Not confident 2- Slightly confident 3- Fairly confident 4- Confident 5- Very confident

**How confident are you in your knowledge of lower extremity musculoskeletal anatomy?**

1- Not confident 2- Slightly confident 3- Fairly confident 4- Confident 5- Very confident

**How confident are you in your knowledge of basic ultrasonography?**

1- Not confident 2- Slightly confident 3- Fairly confident 4- Confident 5- Very confident

**How confident are you that you can apply your knowledge of upper and lower extremity anatomy in a clinical setting?**

1- Not confident 2- Slightly confident 3- Fairly confident 4- Confident 5- Very confident

**How confident are you at identifying the femoral nerve during an ultrasound scanning of a patient in a clinical setting?**

1- Not confident 2- Slightly confident 3- Fairly confident 4- Confident 5- Very confident

**How confident are you at identifying the sciatic nerve during an ultrasound scanning of a patient in a clinical setting?**

1- Not confident 2- Slightly confident 3- Fairly confident 4- Confident 5- Very confident

**How confident are you at identifying the tibial and common peroneal nerves in the Popliteal Block during an ultrasound scanning of a patient in a clinical setting?**

1- Not confident 2- Slightly confident 3- Fairly confident 4- Confident 5- Very confident

**How confident are you at identifying the tibial nerve at the ankle during an ultrasound scanning of a patient in a clinical setting?**

1- Not confident 2- Slightly confident 3- Fairly confident 4- Confident 5- Very confident

**How confident are you at identifying the C5, C6, and C7 Ventral Rami in the Interscalene Block during an ultrasound scanning of a patient in a clinical setting?**

1- Not confident 2- Slightly confident 3- Fairly confident 4- Confident 5- Very confident

**How confident are you at identifying the Trunks of the Brachial Plexus in the Supraclavicular Block during an ultrasound scanning of a patient in a clinical setting?**

1- Not confident 2- Slightly confident 3- Fairly confident 4- Confident 5- Very confident

**How confident are you at identifying the Lateral, Medial, and Posterior Cords in the Infraclavicular Block during an ultrasound scanning of a patient in a clinical setting?**

1- Not confident 2- Slightly confident 3- Fairly confident 4- Confident 5- Very confident

**How confident are you at identifying the Median, Musculocutaneous, Ulnar, and Radial Nerves in an Axillary Block during an ultrasound scanning of a patient in a clinical setting?**

1- Not confident 2- Slightly confident 3- Fairly confident 4- Confident 5- Very confident

**How confident are you in your knowledge of ultrasound anatomy for the upper extremity?**

1- Not confident 2- Slightly confident 3- Fairly confident 4- Confident 5- Very confident

**How confident are you in your knowledge of ultrasound anatomy for the lower extremity?**

1- Not confident 2- Slightly confident 3- Fairly confident 4- Confident 5- Very confident

**POST SURVEY**

**PGY Year _______ Unique ID _____________________**

**(Confidence in musculoskeletal regional anatomy and ultrasound knowledge)**

**How confident are you in your knowledge of upper extremity musculoskeletal anatomy?**

1- Not confident 2- Slightly confident 3- Fairly confident 4- Confident 5- Very confident

**How confident are you in your knowledge of lower extremity musculoskeletal anatomy?**

1- Not confident 2- Slightly confident 3- Fairly confident 4- Confident 5- Very confident

**How confident are you in your knowledge of basic ultrasonography?**

1- Not confident 2- Slightly confident 3- Fairly confident 4- Confident 5- Very confident

**How confident are you that you can apply your knowledge of upper and lower extremity anatomy in a clinical setting?**

1- Not confident 2- Slightly confident 3- Fairly confident 4- Confident 5- Very confident

**How confident are you at identifying the femoral nerve during an ultrasound scanning of a patient in a clinical setting?**

1- Not confident 2- Slightly confident 3- Fairly confident 4- Confident 5- Very confident

**How confident are you at identifying the sciatic nerve during an ultrasound scanning of a patient in a clinical setting?**

1- Not confident 2- Slightly confident 3- Fairly confident 4- Confident 5- Very confident

**How confident are you at identifying the tibial and common peroneal nerves in the Popliteal Block during an ultrasound scanning of a patient in a clinical setting?**

1- Not confident 2- Slightly confident 3- Fairly confident 4- Confident 5- Very confident

**How confident are you at identifying the tibial nerve at the ankle during an ultrasound scanning of a patient in a clinical setting?**

1- Not confident 2- Slightly confident 3- Fairly confident 4- Confident 5- Very confident

**How confident are you at identifying the C5, C6, and C7 Ventral Rami in the Interscalene Block during an ultrasound scanning of a patient in a clinical setting?**

1- Not confident 2- Slightly confident 3- Fairly confident 4- Confident 5- Very confident

**How confident are you at identifying the Superior, Middle, and Inferior Trunks in the Supraclavicular Block during an ultrasound scanning of a patient in a clinical setting?**

1- Not confident 2- Slightly confident 3- Fairly confident 4- Confident 5- Very confident

**How confident are you at identifying the Lateral, Medial, and Posterior Cords in the Infraclavicular Block during an ultrasound scanning of a patient in a clinical setting?**

1- Not confident 2- Slightly confident 3- Fairly confident 4- Confident 5- Very confident

**How confident are you at identifying the Median, Musculocutaneous, Ulnar, and Radial Nerves in an Axillary Block during an ultrasound scanning of a patient in a clinical setting?**

1- Not confident 2- Slightly confident 3- Fairly confident 4- Confident 5- Very confident

**How confident are you in your knowledge of ultrasound anatomy for the upper extremity?**

1- Not confident 2- Slightly confident 3- Fairly confident 4- Confident 5- Very confident

**How confident are you in your knowledge of ultrasound anatomy for the lower extremity?**

1- Not confident 2- Slightly confident 3- Fairly confident 4- Confident 5- Very confident

**How clear were the course objectives?**

1- Not clear 2- Slightly clear 3- Fairly clear 4- Clear 5- Very clear

**How appropriate was the content to your level of learning?**

1- Not appropriate 2- Slightly appropriate 3- Fairly appropriate 4- Appropriate 5- Very appropriate

**How effective was this course in enhancing your knowledge of upper extremity anatomy?**

1- Not effective 2- Slightly effective 3- Fairly effective 4- Effective 5- Very effective

**How effective was this course in enhancing your knowledge of lower extremity anatomy?**

1- Not effective 2- Slightly effective 3- Fairly effective 4- Effective 5- Very effective

**How useful did you find the course overall?**

1- Not useful 2- Slightly useful 3- Fairly useful 4- Useful 5- Very useful

**How useful was the lecture component of the course?**

1- Not useful 2- Slightly useful 3- Fairly useful 4- Useful 5- Very useful

**How useful was the standardized patient component of the course?**

1- Not useful 2- Slightly useful 3- Fairly useful 4- Useful 5- Very useful

**How useful was the cadaveric lab component of the course?**

1- Not useful 2- Slightly useful 3- Fairly useful 4- Useful 5- Very useful

**How useful was this course in comparison to your prior educational experiences?**

1- Not useful 2- Slightly useful 3- Fairly useful 4- Useful 5- Very useful

**What did you find the most useful about this course?**

**What did you find to be the least useful aspect of this course?**

**How can we make the course more useful? Please be as specific as possible.**

**Is there another topic in your discipline that you would like to see a course specifically developed around? Please elaborate.**
